# Managing a Pseudo-Mobitz Type II Atrioventricular (AV) Block Intrapartum: A Case Report

**DOI:** 10.7759/cureus.50221

**Published:** 2023-12-09

**Authors:** Mohd Khairi Othman, Muhammad Zulkifli Konok, Engku Husna Engku Ismail, Zurkurnai Yusof, W Yus Haniff W Isa

**Affiliations:** 1 Department of Internal Medicine, School of Medical Sciences, Universiti Sains Malaysia, Kubang Kerian, MYS; 2 Department of Internal Medicine, Hospital Universiti Sains Malaysia, Kubang Kerian, MYS; 3 Department of Obstetrics & Gynaecology, Hospital Universiti Sains Malaysia, Kubang Kerian, MYS

**Keywords:** transcutaneous pacing, pseudo mobitz type 2, parasympathetic tone, high risk obstetrics, hypervagatonia, mobitz type 2 av block

## Abstract

Atrioventricular (AV) block in pregnancy is rare, but it is a serious arrhythmia that needs to be carefully managed in pregnancy. However, as of now, there are no clear guidelines or consensus for intrapartum management. Most of the time, an intrapartum AV block is secondary to hypervagatonic sinus node dysfunction and is treated conservatively. Hypervagatonic sinus node dysfunction has a heterogeneous presentation of AV block, and pseudo-Mobitz type II in labor is rarely reported. We report a case of pseudo-Mobitz type II AV block during pregnancy due to labor pain, which is successfully managed conservatively.

## Introduction

Atrioventricular (AV) block can occur due to increased intracranial pressure, drugs, infiltrative heart disease, or vasovagal response [1]. Vagal-mediated AV block is a paroxysmal first-, second-, or third-degree AV block associated with slowing the sinus rate. It has a heterogeneous presentation of AV block, which is Mobitz type I, pseudo-Mobitz type II, 2:1 AV block, advanced degree, or complete AV block [2]. Pseudo-Mobitz type II due to vagal meditation is defined as abrupt AV block without PR prolongation [2,3]. Currently, there is no consensus or guidelines available to manage patients with AV block during pregnancy. Most of the time, pseudo-Mobitz type II will be treated conservatively [4]. Recognizing a vagal-mediated AV block intrapartum is important, as it will help management during labor and avoid unnecessary intervention.

## Case presentation

**Figure 1:**
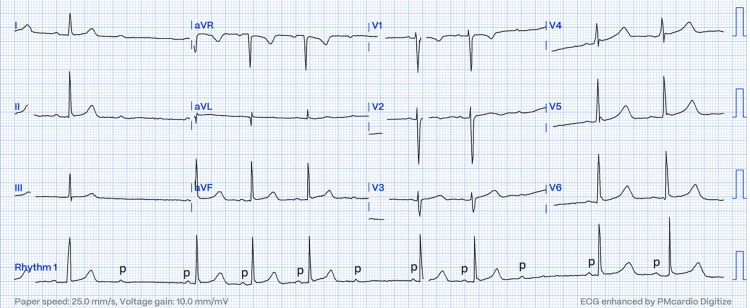
12-lead ECG showed an abrupt, non-conducted P wave (labeled 'p') after a normal PR interval in the previous beat

**Figure 2:**
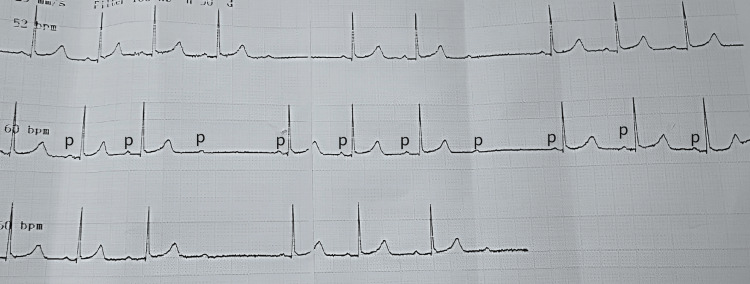
Long lead II showed an abrupt, non-conducted P wave (labeled 'p') in bradycardia with a normal PR interval in the previous beat

A 35-year-old gravida 2 and para 1 presented to our center with a complaint of labor pain, which increased in intensity and frequency. She had a history of maternal tachycardia secondary to subclinical hyperthyroidism in which no treatment was given. Upon examination, her blood pressure was 115/76 mmHg, pulse rate was 50 bpm, and temperature was 37°C. Cardiovascular and respiratory examinations were normal. Abdominal examination was consistent with a gravid uterus, non-tender on palpation, and contractions occurred at a frequency of 2 in 10 minutes. Vaginal examination revealed an os dilation of 3 cm. An ECG was performed, as she had bradycardia, and it showed intermittent 2:1 AV block (Figures 1, 2).

Full blood count renal and liver function test was within normal range. Thyroid function test reveals within normal range.

**Figure 3:**
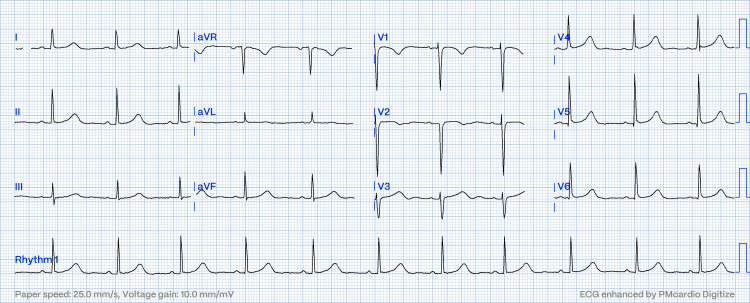
12-lead ECG showed a sinus rhythm with a normal PR interval; resolved pseudo-Mobitz type II AV block

Transthoracic echocardiography showed normal left ventricle size, LVEF 62%. No other abnormalities were detected. She was allowed for spontaneous vaginal delivery with transcutaneous pacing in demand mode. Apart from that, bupivacaine was administered epidurally for pain control. After 30 minutes in labor, she underwent an emergency cesarean section in view of fetal distress. The heart rate improved to above 60 bpm post-delivery, and the ECG showed sinus rhythm (Figure 3).

The patient recovered well postoperatively and was discharged well at day 3 postpartum. During outpatient follow-up at three weeks postpartum, she was well and asymptomatic. Her heart rate returned to her baseline during the follow-up.

## Discussion

AV block in intrapartum is infrequently encountered; hence, there is a lack of evidence or guidelines to manage this patient. AV block intrapartum varies from asymptomatic to symptoms such as dizziness, heart failure, or syncope [5]. Cardiac-related disease in pregnancy has been estimated at 1-2% of the population and carries significant morbidity [6]. Significant physiological changes occur during pregnancy to tolerate the process, which occurs within weeks of conception. Pregnancy is associated with a 15-25% increase in heart rate, which peaks in the third trimester. This phenomenon is associated with an increase in plasma volume by 40% and cardiac output by 30-45% [6]. In terms of ECG changes, pregnancy is associated with cardiac arrhythmias, namely, atrial or ventricular premature complex, supraventricular tachycardia, or atrial fibrillation [7]. AV block during pregnancy is rare and an important condition for early intervention.

The literature reports complete heart block in pregnancy, which was treated with permanent pacemaker implantation [5]. The mechanism of AV block in pregnancy is unknown. However, it has been postulated secondary to atrial stretch due to increased blood volume as pregnancy progresses [1]. Another hypothesis of this condition is an estrogen-mediated effect on intracellular signaling in cardiac myocytes by the GPR30 estrogen receptor [1].

Several causes of AV block need to be looked at in young patients such as congenital heart block, cardiomyopathy, rheumatic fever, infective endocarditis, infiltrative heart disease, drugs, or vagal tone [8]. Our patient did not have any suggestive history as such, which was excluded by blood investigation and echocardiography.

In our patient, the bradycardia is most likely due to hypervagatonic sinus node dysfunction during labor. Labor continuously adjusts cardiac autonomic reflexes by alternate activation of the sympathetic and parasympathetic nervous systems. The vagus nerve, which had right and left branches, was involved in parasympathetic innervation [9]. Overstimulation of this nerve predisposes to bradyarrhythmia. The sympathovagal balance is important to maintain the patient's heart rate during labor. During labor, the high secretion of catecholamines due to pain and anxiety leads to the activation of the sympathetic nervous system; meanwhile, releasing oxytocin leads to parasympathetic nervous system activation [9]. In our patient, the most likely reason for pseudo-Mobitz type II AV block is the excessive release of oxytocin during labor, which stabilized after the delivery. Infrequent wide QRS complex, AV block not initiated by premature beats, presence of PR prolongation, sinus slowing during ventricular asystole, and sinus acceleration on resumption of conduction are criteria for vagal-mediated AV block [2,10].

In managing a patient with heart block in pregnancy, it is important to first stratify patients as asymptomatic and symptomatic. Asymptomatic patients usually do not require any pacing as compared to symptomatic patients. During intrapartum, she was put on a transcutaneous pacemaker in standby mode together with atropine to anticipate worsening AV block during labor. Apart from that, we managed our patient conservatively with good pain control; however, she underwent an emergency cesarean section for fetal distress. Post-delivery, she recovered well, and her heart rate improved to normal sinus rhythm. It proves the hypothesis that the AV block is due to high vagal tone due to high oxytocin levels during labor. For the long-term, a permanent pacemaker is not required for her.

## Conclusions

Pregnancy is a stress condition to the mother especially during labor. Labor pain can stimulate the vagal tone to increase and cause vagal-induced AV block. Recognizing the type of AV block secondary to high vagal tone is important to avoid unnecessary management. In a patient having AV block secondary to high vagal tone, adequate pain control and early anticipation of an emergency cesarean section are important to avoid any maternal morbidity. Using temporary pacing during delivery has been observed to yield benefits; hence, early measures must be taken in a patient who has a high risk of developing AV block intrapartum.
